# The gender gap in self-rated health and education in Spain. A multilevel analysis

**DOI:** 10.1371/journal.pone.0187823

**Published:** 2017-12-07

**Authors:** Sara Pinillos-Franco, Carmen García-Prieto

**Affiliations:** Department of Economic Analysis. University of Valladolid, Valladolid, Spain; La Trobe University, AUSTRALIA

## Abstract

**Background:**

Women tend to report poorer self-rated health than men. It is also well established that education has a positive effect on health. However, the issue of how the benefits of education on health differ between men and women has not received enough attention and the few existing studies which do focus on the subject do not draw a clear conclusion. Therefore, this study aims to analyse whether the positive influence of educational attainment on health is higher for women and whether education helps to overcome the gender gap in self-rated health.

**Methods:**

We analyse cross-sectional data from the 2012 European Union statistics on income and living conditions. We use a logit regression model with odds ratios and a multilevel perspective to carry out a study which includes several individual and contextual control variables. We focused our study on the working population in Spain aged between 25 and 65. The final sample considered is composed of 14,120 subjects: 7,653 men and 6,467 women.

**Results:**

There is a gender gap in self-rated health only for the less educated. This gap is not statistically significant among more highly educated individuals. Attaining a high level of education has the same positive effect on both women’s and men’s self-rated health.

**Conclusions:**

Although we did not find gender disparities when considering the effect of education on health, we show that women’s health is poorer among the less educated, mainly due to labour precariousness and household conditions.

## Introduction

The importance of socioeconomic determinants of health such as income, educational attainment or occupation has been well established [1,2,3,4] although the relationship among them and the causal pathways linking socioeconomics with men’s and women’s health is not yet fully clear.

The special relevance of educational attainment on health has been highlighted by a wide range of studies which have shown that the most highly educated individuals have better self-rated health (SRH) [5] as well as lower morbidity and better mortality rates [6]. This relationship is explained in various ways [7,8,9].

From an individual perspective, higher education, as a human capital endowment [10], is related to higher income and improved working conditions, which have been shown to result in better health [11,12]. Moreover, a higher level of education provides better cognitive skills and access to information, which can lead more highly educated people to have access to better means of improving their health. It is well documented that more highly educated people report a greater sense of control over their lives and, hence, exhibit healthier behaviours [13]. Indeed, less educated people smoke more [14], consume more alcohol [15] and are less physically active than their more highly-educated counterparts.

From a social viewpoint, higher education is related to greater social integration, which provides social support, influence and access to resources, all of which contribute to better individual health [16,17]. It also leads individuals to choose better areas to live, where there is greater access to spaces for physical activity and health care resources, and which curb the possibility of crime and violence [7].

Considering a gender perspective, gender differences in health are well documented. Male mortality rates are higher than female’s although women report more symptoms, use more health care services than men, and tend to report worse SRH [18,19,20,21]. Women’s lower SRH may indicate female socioeconomic disadvantage due to lower income, poorer working conditions, less economic independence, etc. As pointed out before, education may result in better health, although the issue of how the benefits of education on health differ between men and women has received little attention and the few studies that do focus on the subject have thus far failed to yield any clear conclusions. Some recent studies report higher health returns to education for women than men in the USA [22,23], although others find the opposite in Europe [24,25], while some researchers report no statistically significant difference [26,27,28].

The aim of the present paper is to delve deeper into the relationship between gender, education and health. The analysis focuses on the active Spanish population, with SRH being the measure considered to account for the health level of individuals.

## Methods

### Sample selection

European Union statistics on income and living conditions (EU-SILC) provide the reference source for comparative statistics on income distribution and social inclusion in the European Union. In Spain, almost 15,000 private households are selected each year to represent all the private households in the country and all their members aged 16 and over are interviewed. They provide information on household and personal income, education, health, employment, economic deprivation, childcare and household conditions.

A total of 28,210 individuals completed the questionnaires in Spain for the 2012 wave. We only considered those respondents between 25 and 65 years of age who were working either part-time or full-time, or who were unemployed or freelance. From this selection, 288 individuals living in the autonomous cities of Ceuta and Melilla were excluded for reasons of sample homogeneity, as were those who presented missing values in our dependent variable (148 individuals did not declare their health status). Therefore, a total of 14,120 individuals were finally included in our analysis.

### Self-rated health and educational attainment

Health status was measured by individual’s SRH. Indicators of SRH have proved to be good predictors of mortality rates [29,30] although they are multidimensional measures which include different aspects of individuals’ health such as their physical and mental status and are widely used when analysing determinants of health [31].

Respondents were asked to value their own health (How would you rate your health in general?) choosing from among five possible answers: very good, good, fair, bad and very bad. Answers were dichotomized into a dependent variable with two categories: good if the individual’s valuation was very good or good, and bad otherwise. With this variable, we formulated a bivariate logit model.

Individuals’ educational attainment and its influence on their health was studied by considering two categories: lower educated population, which includes those who hold primary or secondary studies or who declared a non-educational background, and higher educated, if they completed tertiary education (mostly university)

### Demographic variables

Among the covariates considered, we included age, splitting the sample into three intervals: from 25 to 40, from 41 to 55, and from 56 to 65 years old. The lower limit is justified by considering that, at that age, individuals have already completed their academic training, and hence, their educational attainment may be measured better.

Gender inequalities were studied by means of a dichotomous variable, and whether the individual was considered an immigrant as a result of having been born outside the country was also taken into account.

### Socioeconomic variables

In order to consider an individual’s situation in the job market, we split respondents into four categories: freelance, part-time worker, full-time worker, and unemployed. Part-time workers were also split into two additional groups, depending on the reason why individuals work less than 30 hours per week. They can either be forced into this type of contract for various reasons (such as studies or training commitments, sickness, housework or because they cannot find a full-time job), or may opt for such employment of their own accord.

Individuals’ income was computed by calculating their equivalised income, according to the so-called modified OECD equivalence scale. Moreover, a special disfavoured economic situation of the household was taken into account with the variable “material deprivation”. Household composition was also analysed as was whether individuals belong to a family containing economically dependent members.

### Contextual variables

Certain elements concerning where individuals live may determine the final impact of their personal factors on their health [32]. Introducing contextual characteristics ensures that we do not lapse into any ecological and atomistic fallacies [33] when drawing inferences. With this aim, the degree of urbanization of the location where individuals live was included in the analysis, since low populated areas tend to lack certain basic facilities such as primary health-care centres and hospitals, in addition to which accessibility to them may prove more difficult. A further negative influence may be the presence of noise, pollution, dirt or other environmental problems in the area where they live, in addition to crime or vandalism issues. Hence, we split living areas into two categories: favourable or unfavourable environment.

### Other variables

We took into consideration the lack of health assistance in case of need, whether the individual mentions not being able to visit the doctor on at least one occasion, when necessary, in the past twelve months. This may have been due to cost, waiting lists or travel difficulties or to the respondent having decided to wait until the symptoms disappeared. Delayed and foregone medical care is a good indicator of inequalities in access to health and can be associated with prolonged morbidity and increased severity of illness [34,35]. Recent studies point out that this indicator has increased in Europe in recent years [36].

### Statistical analysis

For the empirical analysis, we used a bivariate logistic regression, reporting the odds ratios and their significance level. We adopted a multilevel analysis due to the hierarchical nature of our data, with two levels: individual and regional, in order to analyse the possible relationship between individuals’ health and the particular characteristics of the region where their place of residence is located [37]. This perspective allows us to distinguish between individual and environmental factors which affect health.

## Results

Table 1 shows that individuals in the selected sample report good health in general (86%), a fact reinforced for higher educated individuals (92%). Moreover, the percentage of men (54%) is higher than women (46%), although the latter have better educational attainment. Approximately half of the individuals are middle-aged (46%), with a full-time job (55%) and have minors in their care (53%). The majority of the sample are of Spanish nationality (90%), have no post-secondary studies (65%), visit the doctor when necessary (93%), and live in a highly urbanized (71%) and favourable (78%) environment. When stratifying the sample by educational level, differences appear with regard to the situation in the labour market. The unemployment rate of less educated individuals (31%) is double that of their more highly-educated counterparts (15%). In addition, the percentage of full-time workers is lower in this group (48%). This may lead to below average income in the group (12.66 versus 20.24 among highly educated individuals) and to them living in less populated areas (34% versus 20%) to a greater extent.

**Table 1:** Descriptive statistics.

Variables	Whole sample	Higher education	Lower education
Self-rated health (good)	86%	92%	82%
Sex:			
Female	46%	52%	42%
Male	54%	48%	58%
Age:			
Between 25 and 40	38%	46%	34%
Between 41 and 55	46%	43%	48%
Between 56 and 65	15%	11%	17%
Nationality:			
Immigrant	10%	8%	11%
Native	90%	92%	89%
Educational attainment:			
Higher education	35%	-	-
Lower education	65%	-	-
Labour condition:			
Freelance	13%	10%	14%
Part-time worker, through choice	1%	1%	1%
Part-time worker, not through choice	6%	6%	6%
Unemployed	25%	15%	31%
Full-time worker	55%	68%	48%
Minors	53%	54%	52%
No minors	47%	46%	48%
Material deprivation	5%	1%	7%
Medical accessibility:			
Unmet need for medical care, not through choice	3%	3%	3%
Unmet need for medical care, through choice	4%	4%	4%
Always visit doctor when necessary	93%	93%	93%
Household income ^a^	15.32	20.24	12.66
Degree of urbanization:			
Low	29%	20%	34%
High	71%	80%	66%
Environment:			
Favourable	78%	77%	78%
Unfavourable	22%	23%	22%

^a^ Mean of the variable

Table 2 presents the odds ratios of the probability of reporting good health among respondents related to their educational attainment. Higher educated individuals are more likely to report good health than less educated individuals in the unadjusted model (OR: 2.52, 95% CI: 2.23–2.83). Nevertheless, the odds ratios related to educational attainment change to 1.67 (95% CI: 1.46–1.90) in the final model when individual and contextual characteristics are introduced into the estimation, although it remains statistically significant.

**Table 2:** Odds ratios (95% intervals) for respondents reporting good self-rated health. Level of educational attainment is adjusted for individual and contextual characteristics.

Independent variables	Unadjusted	Adjusted for age, sex and nationality	Adjusted for age, sex, nationality and labour condition.	Adjusted for age, sex, nationality, labour condition and all other variables.
Higher education	2.52*** (2.23 to 2.83)	2.23*** (1.98 to 2.52)	1.96*** (1.73 to 2.22)	1.67*** (1.46 to 1.90)
Lower education	1.00	1.00	1.00	1.00
Between 25 and 40 years old		1.00	1.00	1.00
Between 41 and 55 years old		0.41*** (0.36 to 0.46)	0.39*** (0.34 to 0.44)	0.36*** (0.32 to 0.42)
Between 56 and 65 years old		0.20*** (0.17 to 0.23)	0.19*** (0.17 to 0.22)	0.19*** (0.16 to 0.22)
Female		0.84** (0.76 to 0.93)	0.88* (0.79 to 0.97)	0.85** (0.77 to 0.95)
Immigrant		0.79** (0.67 to 0.93)	0.85 (0.72 to 1.00)	0.93 (0.78 to 1.10)
Freelance			0.74*** (0.64 to 0.86)	0.82* (0.70 to 0.96)
Part-time worker, through choice			0.94 (0.45 to 1.95)	1.04 (0.49 to 2.21)
Part-time worker, not through choice			0.67*** (0.55 to 0.83)	0.71** (0.57 to 0.87)
Unemployed			0.48*** (0.43 to 0.54)	0.57*** (0.50 to 0.64)
Full-time worker			1.00	1.00
Minors				1.39*** (1.25 to 1.55)
No minors			1.00	1.00
Material deprivation				0.57*** (0.47 to 0.69)
Unmet need for medical care, not through choice				0.31*** (0.25 to 0.39)
Unmet need for medical care, through choice				0.51*** (0.41 to 0.64)
Always go to doctor when necessary				1.00
Household income				1.02*** (1.01 to 1.02)
Low degree of urbanization				0.79*** (0.71 to 0.89)
High degree of urbanization				1.00
Unfavourable environment				0.63*** (0.56 to 0.71)
Favourable environment				1.00
Random-effects variance	0.066	0.079	0.069	0.053
Log likelihood	-5540.6985	-5284.7167	-5211.0123	-5059.1758

***p<0.001

**p<0.01

*p<0.05

Other interesting results can be obtained from the estimations reported in Table 2. In the model adjusted only for personal factors, we found a negative age gradient since, as individuals grow older, the likelihood of them reporting good health decreases (OR: 0.41, 95% CI: 0.36–0.46 and OR: 0.20, 95% CI: 0.17–0.23). Being a woman or an immigrant reduces the likelihood of reporting good health too (OR: 0.84, 95% CI: 0.76–0.93 and OR: 0.79, 95% CI: 0.67–0.93 respectively). When the rest of the covariates are included in the estimation, these results remain fairly stable, except those concerning being an immigrant, the odds ratio for which becomes non-significant.

As for the remaining variables, income displays a positive albeit small gradient (OR: 1.02, 95% CI: 1.01–1.02). In addition, individuals who suffer material deprivation (OR: 0.57, 95% CI: 0.47–0.69) are much less likely to report good health. The odds ratio of unemployed people is also lower (OR: 0.57, 95% CI: 0.50–0.64) as is that of part-time workers, although in the latter case only for those workers who are forced to accept a part-time job but who would like to work on a full-time basis (OR: 0.71, 95% CI: 0.57–0.87). The same effect occurs when individuals live in an unfavourable environment (OR: 0.63, 95% CI: 0.56–0.71), or in a low urbanized location (OR: 0.79, 95% CI: 0.71–0.89). Finally, unmet medical care needs lower the likelihood of reporting good health, no matter why individuals did not visit the doctor, although it was truer to a greater extent if the individual had certain difficulties accessing the doctor.

As the aim of this paper is to investigate the effects of education and gender on health, we performed different estimations, stratifying by educational attainment and sex. Table 3 summarizes the results of the analysis of gender health inequalities at each educational level: higher and lower educated individuals. Regarding less educated individuals, women display a likelihood of around 15% less than men of reporting good health, with the odds ratios being significant in almost all estimated models (unadjusted and adjusted for the different covariates). With regard to the more educated, women show less likelihood of declaring good health than men in all the estimations carried out, although the odds ratios are not statistically significant in any of the cases.

**Table 3:** Odds ratios (95% coefficient interval) of men and women for each model for respondents reporting good health. Sex is adjusted for individual and contextual characteristics and stratified by educational attainment.

Model	Sex	
	Female	Male
***Higher education***		
Unadjusted	0.96 (0.77 to 1.18)	1.00
Adjusted for age and nationality	0.86 (0.69 to 1.06)	1.00
Adjusted for age, nationality and labour condition	0.85 (0.68 to 1.06)	1.00
Adjusted for age, nationality, labour condition, and all other variables	0.84 (0.67 to 1.04)	1.00
***Lower education***		
Unadjusted	0.83** (0.75 to 0.93)	1.00
Adjusted for age and nationality	0.83** (0.74 to 0.93)	1.00
Adjusted for age, nationality and labour condition	0.89 (0.79 to 1.00)	1.00
Adjusted for age, nationality, labour condition, and all other variables	0.85** (0.76 to 0.96)	1.00

**p<0.01

Looking at the disparities between higher and lower educated (detailed results available from the figshare repository at the following URL: https://figshare.com/s/61f663a75e1bc50a83b3), we found that certain labour situations characterized by precariousness, such as working part-time not through choice, and household material deprivation, are only significant vis-à-vis explaining less educated individuals’ health, specifically where women have a higher risk of presenting poor health. This outcome might be due to women attaching greater importance to family and other life dimensions [38] and, hence, tending to choose non-standard jobs in an effort to strike the right work-life balance [39].

Table 4 displays the results of measuring the effect of education on SRH when the sample is stratified by sex. Achieving a higher level of education increases the likelihood of reporting good health more for women than for men (OR: 2.74, 95% CI: 2.32–3.23 and OR: 2.38, 95% CI: 2.00–2.82 respectively) in the unadjusted model. However, when the analysis is controlled by the remaining covariates, differences between men and women disappear. Hence, it may be concluded that the general effect of educational attainment on health is equal for men and women and that they experience a 69% increase in the likelihood of reporting good health when they achieve a higher level of education.

**Table 4:** Odds ratios (95% coefficient interval) of educational attainment for each model for respondents reporting good health. Level of educational attainment is adjusted for individual and contextual characteristics and stratified by sex.

Model	Educational attainment	
	Higher education	Lower education
***Female***		
Unadjusted	2.74*** (2.32 to 3.23)	1.00
Adjusted for age and nationality	2.31*** (1.95 to 2.73)	1.00
Adjusted for age, nationality and labour condition	1.99*** (1.68 to 2.37)	1.00
Adjusted for age, nationality, labour condition, and all other variables	1.69*** (1.41 to 2.04)	1.00
***Male***		
Unadjusted	2.38*** (2.00 to 2.82)	1.00
Adjusted for age and nationality	2.21*** (1.86 to 2.64)	1.00
Adjusted for age, nationality and labour condition	1.99*** (1.66 to 2.38)	1.00
Adjusted for age, nationality, labour condition, and all other variables	1.69*** (1.40 to 2.03)	1.00

***p<0.001

## Discussion

Information concerning the Spanish working population extracted from European Union statistics on income and living conditions (EU-SILC) reveals that higher educated individuals report better health more often than less educated individuals do. Our bivariate logistic analysis, controlling by gender, socioeconomic and contextual variables, and adopting a multilevel perspective (individuals and regions), confirms a significant higher probability of reporting good health for higher educated individuals. It also points to the existence of gender inequalities in health as women show a significantly lower likelihood of reporting good health than men.

The issue of how education affects women’s and men’s health differently has been addressed in the specific literature applying two alternative hypotheses. The resource substitution view suggests that when resources substitute each other, the lack of one will produce a less important negative effect on health when other resources are present [23,40]. Women have fewer socioeconomic resources than men (less economic independence, fewer opportunities for a full-time job, lower authority…). Hence, women’s health will be more favoured than men’s as a result of improved educational attainment, since the presence of educational resources reduces the negative effect of the lack of other resources for women. The opposite view of the reinforced status proposes that socioeconomically favoured individuals obtain greater gains from improvements in their resources, thus amplifying the gap when compared to the less favoured. In this case, the health benefits provided by increased educational attainment will be greater for men, and will further men’s advantage [23,40].

In order to gain deeper insights into the subject, we conducted separate analysis by educational level, and found that although less educated women display a lower likelihood of reporting good health than men, there are no statistical gender differences in health between higher educated men and higher educated women. This result might lead us to accept a confirmation of the former theory, as women show worse health than men when their educational attainment is lower, whilst improving their educational level allows them to overcome the gap.

We carried out a fresh analysis, this time stratified by sex, and found that when educational attainment rises there is a significantly higher increase in the likelihood of reporting good health for women than for men. Nevertheless, this result is only present in the unadjusted model and does not remain when all the socioeconomic and contextual covariates are considered. The final odds ratios of the effect of educational attainment on health encountered for women and men are the same when all control variables are taken into account.

Hence, analysing all the results together, it seems that education has the same direct effect on health for men and women, although at the same time it provides women with an increase in other socioeconomic resources (for instance, it has been shown that higher levels of education lead to reductions in the gender wage gap suffered by women in the job market [41]), reducing men’s advantage and enhancing their health more. Thus, education allows women to overcome the observed gender health gap within the low educated individuals group.

Our analysis has certain limitations. The data source selected to conduct the study fails to provide any information on individuals’ behavioural risk factors such as tobacco and alcohol consumption, exercise, or whether respondents keep to a healthy and balanced diet. Although healthy lifestyles are important determinants when explaining SRH, we decided to carry out the analysis with the EU-SILC as it provides more detailed information than other surveys about the socioeconomic situation of individuals, particularly with regard to personal and household income and social exclusion. This aspect is quite important as regards ascertaining whether precarious work or unemployment and the consequent loss of income might impact on men and women differently, particularly in the current economic crisis.

Some recent papers have focused on the influence of individuals’ socioeconomic background [42,43] and have pointed out that health returns to education depend on socioeconomic origin. They show that the social position of the family with whom individuals live when they are young is crucial vis-à-vis determining their current education level, their health habits and their socioeconomic position. We did not have such information available although it does pose an interesting subject for further research.

Despite these limitations, our study provides a valuable analysis of the influence of educational attainment on gender inequalities in SRH. The work highlights the importance of promoting education, since this raises the general health level of the population and tends to reduce socioeconomic gender inequalities over time.
